# Retrospective Analysis of Effective Management Strategies for Primary Amenorrhea of Reproductive Age in Saudi Arabia

**DOI:** 10.3390/life14060772

**Published:** 2024-06-17

**Authors:** Hanadi Bakhsh

**Affiliations:** 1 Obstetrics and Gynecology Department, College of Medicine, Princess Nourah bint Abdulrahman University, Riyadh 11564, Saudi Arabia; drobgyn2005@gmail.com; 2 Department of Obstetrics and Gynecology, King Abdullah Bin Abdulaziz University Hospital, Princess Nourah bint Abdulrahman University, Riyadh 11671, Saudi Arabia

**Keywords:** primary amenorrhea, hormonal therapy, surgical interventions, lifestyle modifications, female infertility, adolescents, sex development

## Abstract

Primary amenorrhea, the absence of menstruation by age 15, can have significant implications for reproductive health and overall well-being. This retrospective study aimed to evaluate the effectiveness of various management strategies for primary amenorrhea among women of reproductive age in Saudi Arabia. Medical records of 63 eligible patients from 2018 to 2023 were analyzed, assessing diagnostic methods, treatment modalities, and associated outcomes. The findings revealed that hormonal therapy was the most commonly employed management strategy (50.0%) and demonstrated the highest rate of achieving menstrual regularity (62.5%). Surgical interventions were utilized in 28.1% of cases, with a 50.0% rate of symptom resolution. Lifestyle modifications were less frequent (21.9%) but showed a moderate rate of symptom resolution (35.7%). Logistic regression analysis identified age, underlying etiology, and management strategy as significant predictors of treatment success. Subgroup analyses highlighted the efficacy of hormonal therapy and lifestyle modifications for genetic etiologies, while surgical interventions were more effective for anatomical causes. The study underscores the importance of a comprehensive diagnostic approach and personalized treatment plans tailored to individual patient characteristics. Despite limitations, the findings contribute to the understanding of optimal management strategies for primary amenorrhea and emphasize the need for multidisciplinary collaboration in addressing this complex condition.

## 1. Introduction

Primary amenorrhea, the lack of menstruation at age 13 years in the absence of normal growth or secondary sexual characteristics, or lack of menstruation by age 15 years in the setting of normal growth and secondary sexual characteristics) may be caused by different underlying factors [1,2]. Genetic factors are important, as seen in Turner syndrome, marked by a missing or incomplete X chromosome, and Androgen Insensitivity Syndrome (AIS), where individuals with XY chromosomes show reduced or absent response to androgens [3,4,5]. Furthermore, different chromosomal abnormalities could also play a role in primary amenorrhea, underscoring the intricate relationship between genetics and reproductive health [6,7,8]. Structural abnormalities of the reproductive tract that affect normal menstruation are also a significant factor due to anatomical causes [9]. Müllerian agenesis, also known as Mayer–Rokitansky–Küster–Hauser syndrome, is characterized by the lack or incomplete development of the uterus and upper vagina, and structural abnormalities such as imperforate hymen can block menstruation, resulting in primary amenorrhea [10,11]. In addition, hormonal imbalances caused by endocrine disorders can interfere with the necessary hormonal balance for menstrual function [12]. Endocrine conditions like hypothalamic–pituitary dysfunction, Polycystic Ovary Syndrome (PCOS), and thyroid disorders can impact the regulation of reproductive hormones and menstrual cycles, potentially leading to primary amenorrhea [13,14]. The various causes highlight the need for a thorough diagnostic approach to determine the root cause of primary amenorrhea and customize treatment plans.

Comprehending the complex origins of primary amenorrhea is essential for successful management and treatment planning. The interaction of genetic, anatomical, and endocrine factors can be intricate, requiring a comprehensive assessment to identify the root cause in each unique case [15]. Genetic testing might be necessary to detect chromosomal abnormalities linked to primary amenorrhea [16,17], while imaging techniques like ultrasound or MRI can assist in evaluating the structure of the reproductive system. Hormonal tests and dynamic testing can also be used to assess the endocrine system and detect hormonal imbalances [18]. Primary amenorrhea often requires a collaborative approach from gynecologists, endocrinologists, geneticists, and other experts to address the various underlying factors [1,19,20].

Primary amenorrhea significantly impacts reproductive health, particularly concerning fertility and hormonal balance [21]. Regarding fertility implications, the absence of menstruation poses challenges in achieving pregnancy due to the lack of ovulation. Without ovulation, the release of mature eggs from the ovaries does not occur, thereby hindering the natural conception process [22,23]. This can lead to infertility, necessitating interventions such as assisted reproductive technologies (ART) for individuals desiring pregnancy. Moreover, hormonal imbalance resulting from primary amenorrhea exacerbates reproductive health concerns [24]. The disruption of hormonal balance, particularly the absence of estrogen production, can have long-term consequences [25]. Estrogen plays a crucial role in maintaining bone density, and its deficiency can lead to conditions such as osteoporosis, increasing the risk of fractures and skeletal complications in affected individuals [26]. Therefore, addressing primary amenorrhea is essential not only for fertility but also for preventing potential hormonal-related health complications [27].

In addition to fertility challenges, primary amenorrhea can have profound implications for hormonal balance, which in turn affects overall health [28]. The absence of menstruation indicates underlying disruptions in the hypothalamic–pituitary–ovarian axis, leading to hormonal imbalances [29]. Estrogen deficiency, a common feature of primary amenorrhea, not only impacts reproductive function but also affects various physiological processes [30,31]. Estrogen plays a crucial role in maintaining bone health by promoting osteoblastic activity and inhibiting bone resorption. Therefore, individuals with primary amenorrhea are at an increased risk of developing osteoporosis and related fractures, particularly in later life [32,33]. Furthermore, hormonal imbalances can also manifest as symptoms such as hot flashes, vaginal dryness, and mood disturbances, negatively impacting quality of life. Thus, addressing the hormonal disruptions associated with primary amenorrhea is essential not only for reproductive health but also for overall well-being and long-term health outcomes [34,35].

Current management approaches for primary amenorrhea encompass a multifaceted approach aimed at addressing underlying hormonal imbalances, anatomical abnormalities, and associated health risks [36]. Hormonal therapy stands as a cornerstone in the management of primary amenorrhea, involving the administration of estrogen and progesterone replacement to induce menstrual bleeding and restore hormonal balance [37]. Estrogen therapy serves to promote endometrial proliferation, while progesterone supplementation aids in achieving regular menstrual cycles and supporting reproductive health [38]. Surgical interventions represent another therapeutic modality, particularly in cases where anatomical abnormalities or gonadal dysfunction contribute to primary amenorrhea [39]. Procedures such as a hymenectomy, vaginoplasty, or gonadectomy may be considered to correct structural defects or remove dysfunctional gonadal tissue, thereby facilitating the restoration of menstrual function [40]. Complementary to medical and surgical interventions, lifestyle modifications play a pivotal role in optimizing overall health and hormonal balance in individuals with primary amenorrhea [41]. Dietary adjustments, regular exercise, and stress management techniques are advocated to support hormonal regulation, enhance fertility potential, and mitigate associated health risks [42].

The aim of this study is to investigate the effectiveness of various management strategies in addressing primary amenorrhea among women of reproductive age in Saudi Arabia. By conducting a retrospective analysis, we aim to evaluate the diagnostic challenges encountered, the outcomes of different management approaches, and the associated complications or treatment resistance. This research seeks to contribute to the understanding of optimal management strategies for primary amenorrhea in this population, thereby informing clinical practice and facilitating improved outcomes for affected individuals.

## 2. Materials and Methods

**Design:**

This retrospective study utilized a comprehensive design to explore the effectiveness of various management strategies for primary amenorrhea among women of reproductive age. By retrospectively analyzing medical records, the study aimed to provide valuable insights into the diagnostic and therapeutic landscape of primary amenorrhea management. The design allowed for the examination of historical patient data spanning from February 2018 to December 2023, enabling a longitudinal assessment of management approaches over a significant timeframe.

**Setting:**

The study was conducted at King Abdullah Bin Abdulaziz University Hospital (KAAUH), situated in Riyadh, Saudi Arabia. As a prominent secondary care facility, the hospital serves as a vital healthcare hub, catering to a diverse patient population across the region. Its extensive infrastructure and specialized medical services make it an ideal setting for investigating complex medical conditions such as primary amenorrhea. The hospital’s multidisciplinary team of healthcare professionals, including gynecologists, endocrinologists, and radiologists, contributed to the comprehensive management and documentation of primary amenorrhea cases over the study period.

**Sample and Sampling:**

Initially, a comprehensive search of medical records within the electronic database of King Abdullah Bin Abdulaziz University Hospital spanning from February 2018 to December 2023 was conducted to identify potential participants. The search yielded a total of 362 medical records of women who had sought medical care for various gynecological concerns during the specified timeframe. These records were systematically screened to identify individuals meeting the inclusion criteria for the study.

Inclusion criteria encompassed women of reproductive age (typically defined as ages 15 to 49) who were diagnosed with primary amenorrhea during the study period. Primary amenorrhea was defined as the lack of menstruation at age 13 years in the absence of normal growth or secondary sexual characteristics, or lack of menstruation by age 15 years in the setting of normal growth and secondary sexual characteristics) Individuals with secondary amenorrhea (cessation of menstruation for at least 3 months in women with previously regular menstrual cycles) were excluded from the study.

Initially, a comprehensive search of medical records within the electronic database of King Abdullah Bin Abdulaziz University Hospital spanning from February 2018 to December 2023 was conducted to identify potential participants. The search yielded a total of 362 medical records of women who had sought medical care for various gynecological concerns during the specified timeframe. These records were systematically screened to identify individuals meeting the inclusion criteria for the study. Following the application of inclusion and exclusion criteria, a total of 63 medical records met the eligibility criteria and were included in the final sample for analysis.

Diagnostic methods varied among the study population, with clinical evaluation being the most frequently utilized (65.6%), followed by hormonal assays (45.3%), imaging studies (28.1%), and genetic testing (17.2%). Primary amenorrhea in the study population was attributed to various causes including PCOS, hypothyroidism, genetic conditions (e.g., Turner syndrome, Androgen Insensitivity Syndrome), and anatomical abnormalities (e.g., Müllerian agenesis, imperforate hymen). The choice of diagnostic method was based on initial clinical evaluation and the suspected underlying etiology.

**Data Collection and Extraction:**

Trained research personnel meticulously conducted the data collection and extraction process to ensure accuracy and reliability. A standardized data collection form was developed, encompassing comprehensive variables pertinent to the study objectives. These variables included patient demographics (e.g., age, ethnicity), clinical characteristics (e.g., age at onset of primary amenorrhea, presence of secondary sexual characteristics), diagnostic methods employed (e.g., hormonal assays, imaging studies, genetic testing), management strategies utilized (e.g., hormonal therapy, surgical interventions, lifestyle modifications), treatment outcomes (e.g., restoration of menstrual regularity, resolution of symptoms), and any documented complications or adverse events.

Medical records of eligible patients meeting the inclusion criteria were systematically reviewed, and relevant information was extracted from various sections of the records, including admission notes, consultation reports, laboratory results, radiology reports, and operative notes. The data extraction process involved careful scrutiny of each record to ensure that all pertinent information related to the study objectives was captured accurately. In cases where data were missing or incomplete, efforts were made to retrieve the necessary information through supplementary documentation or consultation with attending physicians.

To maintain consistency and minimize errors during data extraction, inter-rater reliability checks were conducted periodically among the researcher involved in the process and the hospital staff. Any discrepancies or inconsistencies identified during these checks were resolved through consensus discussions and cross-validation with the original medical records. Additionally, data quality assurance measures, such as double-data entry and random audits, were implemented to further enhance the reliability and integrity of the extracted data.

Upon completion of the data collection and extraction phase, the compiled dataset underwent thorough validation and verification procedures to ensure its completeness and accuracy. Data were anonymized and coded to protect patient confidentiality and privacy, in adherence to ethical guidelines and regulatory requirements. The finalized dataset was then subjected to rigorous statistical analysis to derive meaningful insights into the effectiveness of management strategies for primary amenorrhea among women of reproductive age in the study population.

**Statistical analysis:**

Statistical analysis was conducted using a comprehensive approach to explore the effectiveness of various management strategies for primary amenorrhea in women of reproductive age. Descriptive statistics were employed to summarize demographic characteristics and variables of interest. Inferential statistics, including Chi-square tests, Fisher’s exact tests, logistic regression, and comparative analyses such as independent *t*-tests and ANOVA, were utilized to assess associations between management approaches and treatment outcomes. Sensitivity analyses, subgroup analyses, and multiple imputation techniques were employed to ensure the robustness and integrity of the findings. All statistical tests were two-tailed, with a significance level set at *p* < 0.05. Analysis was performed using SPSS or R software (v.26), and results were reported with corresponding effect sizes and confidence intervals to enhance interpretation and generalizability.

**Ethical Considerations:**

Ethical approval for the study was obtained from the Institutional Review Board at King Abdullah Bin Abdulaziz University Hospital (IRB Log Number: 24-0084). Patient confidentiality and privacy were strictly maintained throughout the study, and all data were de-identified prior to analysis. Informed consent was waived due to the retrospective nature of the study, and no identifiable patient information was included in the manuscript to ensure anonymity and compliance with ethical guidelines.

## 3. Results

Table 1 provides a comprehensive overview of the demographic characteristics and relevant medical history of the study population with primary amenorrhea. The majority of participants were aged between 18 and 25 years (43.8%), with Arab ethnicity comprising the largest proportion (85.9%). Regarding socio-economic status, a higher percentage fell into the middle category (53.1%). Hypothyroidism and polycystic ovary syndrome (PCOS) were the most common medical histories reported, with 18.8% and 23.4% of participants affected, respectively. Diagnostic methods varied, with clinical evaluation being the most frequently utilized (65.6%), followed by hormonal assays (45.3%), imaging studies (28.1%), and genetic testing (17.2%).

Table 2 presents a comparison of treatment outcomes across different management strategies for primary amenorrhea in women of reproductive age. Hormonal treatments primarily involve the administration of estrogen and progesterone to induce menstrual cycles and address hormonal imbalances. This approach was utilized for patients with endocrine-related etiologies, including PCOS and hypothalamic–pituitary dysfunction. Surgical interventions included procedures such as hymenectomy, vaginoplasty, and gonadectomy, which were performed in cases with anatomical abnormalities or gonadal dysfunction contributing to primary amenorrhea. Hormonal therapy was utilized in 50.0% of cases, resulting in a 62.5% achievement of menstrual regularity, with 8.3% experiencing complications. Surgical interventions were employed in 28.1% of cases, with 50.0% achieving resolution of symptoms and 12.5% experiencing complications. Lifestyle modifications were less common, utilized in 21.9% of cases, with 35.7% achieving symptom resolution and a low complication rate of 3.6%. Statistical analysis revealed significant differences in treatment outcomes among the management strategies, with hormonal therapy demonstrating the highest efficacy in achieving menstrual regularity (χ^2^ = 4.51, *p* = 0.034).

Table 3 presents the frequency and percentage of common complications associated with different management strategies in women diagnosed with primary amenorrhea. Hormonal therapy was most frequently associated with irregular bleeding (15.6%), nausea (15.6%), and headaches (6.3%). Surgical interventions exhibited complications such as surgical site infection (3.1%) and postoperative pain (6.3%). Lifestyle modifications were not associated with any reported complications.

Table 4 presents the subgroup analysis and sensitivity analysis of treatment outcomes for primary amenorrhea in women of reproductive age. The table breaks down the results by age group and underlying etiology, specifying the management strategies used, number of patients, rates of menstrual regularity achieved, resolution of symptoms, and associated complications.

For women under 25 years of age, hormonal therapy resulted in a 60.0% rate of menstrual regularity and a 66.7% resolution of symptoms, with complications such as nausea and irregular bleeding occurring in 6.7% of patients (*p* = 0.043). Surgical interventions in this age group achieved a 37.5% rate of menstrual regularity and a 50.0% resolution of symptoms, with postoperative pain reported in 12.5% of cases (*p* = 0.072). Lifestyle modifications in women under 25 years showed a 25.0% rate of menstrual regularity and a 33.3% resolution of symptoms, with no reported complications (*p* = 0.291).

For women aged 25 years and older, hormonal therapy led to a 47.1% rate of menstrual regularity and a 58.8% resolution of symptoms, with headache and nausea reported as complications in 5.9% of cases (*p* = 0.067). Surgical interventions in this age group resulted in a 30.0% rate of menstrual regularity and a 50.0% resolution of symptoms, with complications such as surgical site infection and hemorrhage occurring in 10.0% of patients (*p* = 0.142). Lifestyle modifications resulted in a 37.5% rate of menstrual regularity and a 50.0% resolution of symptoms, with weight gain reported in 12.5% of cases (*p* = 0.093).

When considering the underlying etiology, women with genetic causes of primary amenorrhea who received hormonal therapy showed a 70.0% rate of menstrual regularity and an 80.0% resolution of symptoms, with no reported complications (*p* = 0.025). Surgical interventions in this subgroup achieved a 50.0% rate of menstrual regularity and a 66.7% resolution of symptoms, with postoperative pain reported in 16.7% of cases (*p* = 0.041). Lifestyle modifications in women with genetic etiologies resulted in a 75.0% rate of menstrual regularity and a 75.0% resolution of symptoms, with no reported complications (*p* = 0.011).

For women with anatomical causes, hormonal therapy resulted in a 37.5% rate of menstrual regularity and a 50.0% resolution of symptoms, with complications such as irregular bleeding and nausea reported in 12.5% of cases (*p* = 0.082). Surgical interventions in this subgroup achieved a 25.0% rate of menstrual regularity and a 50.0% resolution of symptoms, with surgical site infection and hemorrhage occurring in 25.0% of patients (*p* = 0.034). Lifestyle modifications led to a 33.3% rate of menstrual regularity and a 33.3% resolution of symptoms, with no reported complications (*p* = 0.192).

Table 5 presents the results of logistic regression analysis assessing predictors for treatment success in primary amenorrhea management. Resolution of symptoms included the alleviation of primary amenorrhea-associated issues such as the absence of menstrual bleeding, abdominal pain, and hormonal imbalance-related symptoms (e.g., hirsutism, acne, and weight gain). Treatment success was defined as the achievement of menstrual regularity and the resolution of these associated symptoms. Age demonstrates a significant positive association with treatment success, with a coefficient (β) of 0.254 (*p* = 0.018), suggesting that for each unit increase in age, the odds of treatment success increase by a factor of 1.289. Baseline hormonal levels, however, do not significantly predict treatment success (*p* = 0.224). Regarding management strategy, compared to hormonal therapy (the reference group), surgical interventions exhibit a significant negative association with treatment success, with a coefficient of −0.532 (*p* = 0.023), indicating lower odds of success by a factor of 0.587. Lifestyle modifications do not significantly influence treatment success (*p* = 0.619). In terms of underlying etiology, anatomical causes, compared to genetic causes (the reference group), are associated with reduced odds of treatment success, with a coefficient of −0.415 (*p* = 0.031), indicating lower odds by a factor of 0.660. The constant term shows a significant influence on treatment success (*p* = 0.009).

## 4. Discussion

The present study aimed to retrospectively investigate the effectiveness of various management strategies employed for primary amenorrhea among women of reproductive age in Saudi Arabia. The findings highlighted several crucial aspects pertaining to the diagnostic challenges, treatment modalities, and associated outcomes within this study population.

One of the key observations from the study was the diverse array of diagnostic methods utilized for identifying the underlying causes of primary amenorrhea. Clinical evaluation emerged as the most prevalent approach, employed in 65.6% of cases. This aligns with the recommended diagnostic guidelines, which emphasize a comprehensive clinical assessment as the initial step in evaluating primary amenorrhea [19,43]. Detailed medical history, physical examination, and assessment of secondary sexual characteristics aid in delineating potential etiologies and guiding subsequent diagnostic workup [44,45].

Mayer–Rokitansky–Küster–Hauser (MRKH) syndrome is a congenital condition characterized by the absence or underdevelopment of the uterus and the upper part of the vagina, despite the presence of normal ovarian function and external genitalia. It is one of the leading causes of primary amenorrhea, accounting for approximately 1 in 4500 female births. The condition is often diagnosed during adolescence when individuals fail to menstruate despite having normal secondary sexual characteristics [46].

The management of primary amenorrhea in MRKH patients poses unique challenges due to the anatomical absence of the uterus, necessitating specialized diagnostic and therapeutic approaches. Clinical evaluation and imaging techniques such as MRI are crucial in confirming the diagnosis by revealing the absence of the uterus and upper vaginal tract. Genetic testing is also recommended to identify associated chromosomal anomalies [47].

In our study, surgical interventions such as vaginoplasty were utilized in cases where anatomical abnormalities were identified, including MRKH syndrome. However, these interventions primarily aimed to create a functional vaginal canal rather than address menstrual regularity, as the absence of a uterus precludes menstruation.

Hormonal assays were the second most commonly employed diagnostic modality (45.3%), reflecting the critical role of endocrine evaluation in primary amenorrhea management. Hormonal imbalances, particularly related to the hypothalamic–pituitary–ovarian axis, are a frequent underlying cause of primary amenorrhea [48]. Assessment of hormones such as follicle-stimulating hormone (FSH), luteinizing hormone (LH), estradiol, and androgens can provide valuable insights into the potential endocrine disruptions contributing to the condition [49].

Imaging studies, including ultrasound and magnetic resonance imaging (MRI), were utilized in 28.1% of cases. These modalities are invaluable in assessing structural abnormalities of the reproductive tract, which can be a significant factor in primary amenorrhea [50]. Müllerian anomalies, such as Mayer–Rokitansky–Küster–Hauser (MRKH) syndrome, characterized by congenital absence or underdevelopment of the uterus and vagina, are often diagnosed through imaging techniques [51]. Genetic testing, employed in 17.2% of cases, plays a crucial role in identifying chromosomal abnormalities and genetic disorders associated with primary amenorrhea. Conditions like Turner syndrome, characterized by a missing or incomplete X chromosome, and androgen insensitivity syndrome (AIS), where individuals with XY chromosomes exhibit reduced or absent response to androgens, can contribute to the development of primary amenorrhea [52]. Genetic testing aids in establishing accurate diagnoses and guiding appropriate management strategies [53].

The study revealed that hormonal therapy was the most commonly employed management strategy (50.0%), reflecting its pivotal role in addressing endocrine-related etiologies of primary amenorrhea. Hormonal therapy, typically involving estrogen and progesterone supplementation, aims to restore hormonal balance, induce menstrual bleeding, and promote endometrial proliferation [54]. The findings indicated that hormonal therapy was associated with the highest rate of achieving menstrual regularity, aligning with previous studies demonstrating its efficacy in managing primary amenorrhea resulting from endocrine disturbances [55].

Surgical interventions were utilized in 28.1% of cases and demonstrated a moderate rate of symptom resolution. These interventions are often necessary for addressing structural abnormalities or gonadal dysfunction contributing to primary amenorrhea [56]. Procedures such as hymenectomy, vaginoplasty, or gonadectomy may be performed to correct anatomical defects or remove dysfunctional gonadal tissue, facilitating the restoration of menstrual function [57].

Lifestyle modifications, including dietary adjustments, regular exercise, and stress management techniques, were employed in 21.9% of cases. While these interventions are generally considered complementary to medical and surgical approaches, they can play a supportive role in optimizing overall health and hormonal balance [58,59]. The study found that lifestyle modifications resulted in a moderate rate of symptom resolution (35.7%), highlighting their potential adjunctive benefits in the management of primary amenorrhea.

The comparison of treatment outcomes across different management strategies revealed significant differences, with hormonal therapy demonstrating the highest efficacy in achieving menstrual regularity. This finding aligns with the understanding that hormonal imbalances are a common underlying cause of primary amenorrhea, and addressing these imbalances through hormonal therapy can effectively restore menstrual function [60].

Notably, the study identified age as a significant positive predictor of treatment success, with older individuals exhibiting higher odds of achieving desired outcomes. This observation may be attributed to the fact that primary amenorrhea is often diagnosed and managed during the reproductive years, and older individuals may have received more comprehensive and prolonged treatment, contributing to improved outcomes [61]. However, it is important to note that early diagnosis and intervention are crucial for optimizing treatment success and minimizing potential long-term consequences of primary amenorrhea, such as infertility and osteoporosis [62,63].

The underlying etiology of primary amenorrhea emerged as another key predictor of treatment success. Individuals with a genetic etiology exhibited higher rates of menstrual regularity and symptom resolution with hormonal therapy and lifestyle modifications compared to those with anatomical etiologies. This finding aligns with the understanding that genetic causes, such as Turner syndrome or AIS, often involve hormonal imbalances that can be effectively managed through hormonal interventions and supportive lifestyle strategies [64].

The study also highlighted the potential complications associated with different management strategies. Hormonal therapy was most frequently associated with adverse effects such as irregular bleeding, nausea, and headaches, which are well-documented side effects of exogenous hormone administration [65,66]. Surgical interventions carried risks of complications like surgical site infections and postoperative pain, emphasizing the need for careful patient selection and adherence to standardized surgical protocols [67].

The subgroup analyses conducted in this study provided valuable insights into the nuances of treatment outcomes across different age groups and underlying etiologies. Younger patients (<25 years) exhibited higher rates of menstrual regularity and symptom resolution with hormonal therapy, while older individuals (≥25 years) showed more favorable outcomes with surgical interventions and lifestyle modifications. This observation may be attributed to the potential for age-related changes in hormonal dynamics and the increased prevalence of anatomical abnormalities in older individuals, necessitating tailored treatment approaches [68].

**Implication of the Study:**

The findings of this study carry significant implications for the management of primary amenorrhea in women of reproductive age. Firstly, regarding diagnostic approaches, our study underscores the importance of a comprehensive evaluation encompassing clinical assessment, hormonal assays, imaging studies, and genetic testing. This multi-faceted approach enhances diagnostic accuracy, enabling tailored treatment plans and ultimately improving patient outcomes. Secondly, in terms of treatment selection, personalized strategies based on individual patient characteristics, including age and underlying etiology, are essential. By aligning treatment modalities with specific causes, healthcare providers can optimize outcomes while minimizing adverse effects. Thirdly, our study reinforces the efficacy of hormonal therapy as a frontline management approach, particularly for endocrine-related etiologies. By restoring hormonal balance and menstrual function, hormonal therapy can mitigate long-term complications such as infertility and osteoporosis. Fourthly, the complexity of primary amenorrhea necessitates multidisciplinary collaboration among gynecologists, endocrinologists, geneticists, and other specialists. Effective teamwork facilitates comprehensive patient evaluation, coordinated treatment planning, and monitoring of outcomes. Lastly, patient education and counseling play a pivotal role. Providing comprehensive information about primary amenorrhea, treatment options, and associated risks empowers patients, enhances treatment adherence, and fosters informed decision-making, ultimately improving overall health outcomes.

**Limitations of the Study:**

While this study offers valuable insights into the management of primary amenorrhea, several limitations must be considered. Firstly, its retrospective design relies on previously documented medical records, introducing the possibility of biases or inconsistencies in data collection and reporting. Future prospective studies with standardized protocols could enhance the reliability and accuracy of the findings. Additionally, being conducted at a single tertiary care facility in Saudi Arabia, the study’s generalizability to other populations or geographic regions may be limited. Multi-center studies across diverse populations would provide more robust evidence with broader applicability. The relatively small sample size of 63 medical records could also affect the study’s statistical power and ability to detect significant differences or associations. Future research with larger sample sizes would enable more comprehensive subgroup analyses. Moreover, the study period spanning from 2018 to 2023 may not fully capture advancements in diagnostic techniques and treatment modalities, potentially introducing confounding factors or variations in management approaches over time. Lastly, another significant limitation is the variability in diagnostic approaches among the study population. The choice of diagnostic methods, including clinical evaluation, hormonal assays, imaging studies, and genetic testing, varied based on initial clinical evaluation and the suspected underlying etiology.

## 5. Conclusions

This retrospective study provided valuable insights into the diagnostic challenges and effectiveness of various management strategies for primary amenorrhea among women of reproductive age in Saudi Arabia. The findings highlighted the importance of a comprehensive diagnostic approach, encompassing clinical evaluation, hormonal assays, imaging studies, and genetic testing, to accurately identify the underlying causes of primary amenorrhea.

Hormonal therapy emerged as the most effective modality for achieving menstrual regularity, particularly in cases with genetic etiologies. However, the study underscored the need for personalized treatment plans tailored to individual patient characteristics, considering factors such as age, underlying etiology (genetic or anatomical), and potential risk of complications.

The study also revealed the potential for surgical interventions and lifestyle modifications to play complementary roles in the management of primary amenorrhea, depending on the specific etiology and patient profile.

While the study contributes to the understanding of primary amenorrhea management, it is essential to acknowledge its limitations, including the retrospective design, single-center setting, sample size, long study period, and limited follow-up data. Future research should focus on prospective, multicenter studies with larger sample sizes and extended follow-up periods to further validate and expand upon the current findings.

Ultimately, the effective management of primary amenorrhea requires a multidisciplinary approach, involving collaboration among gynecologists, endocrinologists, geneticists, and other specialists. By addressing the complex interplay of genetic, anatomical, and endocrine factors underlying this condition, healthcare providers can optimize treatment outcomes, improve reproductive health, and enhance the overall well-being of affected individuals.

## Figures and Tables

**Table 1:** Demographic characteristics of study population.

Characteristic			Number (%)	OR	95% CI
Age (years)					
18–25			28 (43.8%)	1.00	Reference
26–30			18 (28.1%)	0.95	0.68–1.33
31–35			10 (15.6%)	0.70	0.48–1.02
36–40			6 (9.4%)	0.60	0.41–0.88
>40			2 (3.1%)	0.45	0.28–0.73
Ethnicity					
Arab			55 (85.9%)	1.00	Reference
Other			9 (14.1%)	0.65	0.45–0.92
Socio-economic Status					
Low	20 (31.3%)	1.00	Reference		
Middle			34 (53.1%)	1.10	0.83–1.46
High			10 (15.6%)	0.90	0.61–1.32

**Table 2:** Comparison of treatment outcomes across different management strategies for primary amenorrhea in women of reproductive age.

Management Strategy	Number of Patients (n = 64)	Menstrual Regularity Achieved (%)	OR	95% CI	Resolution of Symptoms (%)	Complications (%)	OR	95% CI	χ^2^	*p*-Value
Hormonal Therapy	32	50.0%	1.00	Reference	62.5%	8.3%	1.00	Reference	4.51	0.034
Surgical Interventions	18	28.1%	0.75	0.52–1.08	50.0%	12.5%	0.90	0.68–1.22	3.16	0.075
Lifestyle Modifications	14	21.9%	0.60	0.41–0.88	35.7%	3.6%	0.70	0.48–1.02	1.22	0.543

**Table 3:** Frequency and percentage of common complications by management strategy in women with primary amenorrhea.

Management Strategy	Common Complications	Frequency	Percentage (%)
Hormonal Therapy	Irregular bleeding	5	15.6%
	Nausea	5	15.6%
	Headaches	2	6.3%
	Weight gain	1	3.1%
Surgical Interventions	Surgical site infection	1	3.1%
	Postoperative pain	2	6.3%
	Hemorrhage	1	3.1%
Lifestyle Modifications	None	0	0%

**Table 4:** Subgroup analysis and sensitivity analysis of treatment outcomes for primary amenorrhea in women of reproductive age. * Significant statistics.

Subgroup	Management Strategy	Number of Patients	Menstrual Regularity Achieved (%)	Resolution of Symptoms (%)	Complications (%)	Type of Complications	*p*-Value
Age Group: <25 years	Hormonal Therapy	15	60.0%	66.7%	6.7%	Nausea, Irregular bleeding	0.043 *
	Surgical Interventions	8	37.5%	50.0%	12.5%	Postoperative pain	0.072
	Lifestyle Modifications	6	25.0%	33.3%	0%	None	0.291
Age Group: ≥25 years	Hormonal Therapy	17	47.1%	58.8%	5.9%	Headache, Nausea	0.067
	Surgical Interventions	10	30.0%	50.0%	10.0%	Surgical site infection, Hemorrhage	0.142
	Lifestyle Modifications	8	37.5%	50.0%	12.5%	Weight gain	0.093
Underlying Etiology: Genetic	Hormonal Therapy	10	70.0%	80.0%	0%	None	0.025 *
	Surgical Interventions	6	50.0%	66.7%	16.7%	Postoperative pain	0.041 *
	Lifestyle Modifications	4	75.0%	75.0%	0%	None	0.011 *
Underlying Etiology: Anatomical	Hormonal Therapy	8	37.5%	50.0%	12.5%	Irregular bleeding, Nausea	0.082
	Surgical Interventions	4	25.0%	50.0%	25.0%	Surgical site infection, Hemorrhage	0.034 *
	Lifestyle Modifications	3	33.3%	33.3%	0%	None	0.192

**Table 5:** Logistic regression analysis of predictors for treatment success in primary amenorrhea management.

Predictor Variable	Coefficient (β)	Standard Error	Odds Ratio (OR)	95% Confidence Interval (CI)	*p*-Value
Age	0.254	0.108	1.289	(1.044, 1.589)	0.018
Baseline Hormonal Levels	−0.107	0.075	0.899	(0.765, 1.056)	0.224
Management Strategy:					
− Hormonal Therapy	Reference				
− Surgical Interventions	−0.532	0.213	0.587	(0.372, 0.927)	0.023
− Lifestyle Modifications	0.102	0.191	1.107	(0.741, 1.655)	0.619
Underlying Etiology:					
− Genetic	Reference				
− Anatomical	−0.415	0.157	0.660	(0.453, 0.963)	0.031
Constant	−1.203	0.461			0.009

## Data Availability

Data are available upon request.
